# Surveillance for *Clostridium difficile* Infection: ICD-9 Coding Has Poor Sensitivity Compared to Laboratory Diagnosis in Hospital Patients, Singapore

**DOI:** 10.1371/journal.pone.0015603

**Published:** 2011-01-20

**Authors:** Monica Chan, Poh Lian Lim, Angela Chow, Mar Kyaw Win, Timothy M. Barkham

**Affiliations:** 1 Department of Infectious Diseases, Tan Tock Seng Hospital, Singapore, Singapore; 2 Department of Clinical Epidemiology, Tan Tock Seng Hospital, Singapore, Singapore; 3 Department of Laboratory Medicine, Tan Tock Seng Hospital, Singapore, Singapore; Universidade Federal de Minas Gerais, Brazil

## Abstract

**Introduction:**

*Clostridium difficile* infection (CDI) is an increasingly recognized nosocomial infection in Singapore. Surveillance methods include laboratory reporting of *Clostridium difficile* toxin assays (CDTA) or use of International Classification of Diseases, 9^th^ Revision (ICD-9) discharge code 008.45. Previous US studies showed good correlation between CDTA and ICD-9 codes. However, the use of ICD-9 codes for CDI surveillance has not been validated in other healthcare settings.

**Methods:**

We compared CDI rates based on CDTA to ICD-9 codes for all discharges in 2007 from our hospital to determine sensitivity and specificity of ICD-9 codes. Demographic and hospitalization data were analyzed to determine predictors for missing ICD-9 codes.

**Results:**

During 2007, there were 56,352 discharges. Of these, 268 tested CDTA-positive but only 133 were assigned the CDI ICD-9 code. A total of 141 discharges had the ICD-9 code; 8 were CDTA-negative, the rest were CDTA-positive. Community-acquired CDI accounted for only 3.2% of cases. The sensitivity and specificity of ICD-9 codes compared to CDTA were 49.6% and 100% respectively. Concordance between CDTA and ICD-9 codes was 0.649 (p<.001). Comparing concordant patients (CDTA+/ICD9+) to discordant patients (CDTA+/ICD9−), concordant patients were more likely to be over 50 years of age (OR 3.49, 95% CI 1.66–7.34, p = .001) and have shorter time from admission to testing (OR 0.98, 95% CI 0.97–0.99, p = .009).

**Discussion:**

Unlike previous studies in the US, ICD-9 codes substantially underestimate CDI in Singapore compared to microbiological data. Older patients with shorter time to testing were less likely to have missing ICD-9 codes.

## Introduction

*Clostridium difficile* infection (CDI) is an emerging healthcare-associated problem in Singapore. Among hospitalized patients in our institution, CDI incidence has risen 4-fold from 1.49 cases per 10,000 patient-days in 2001 to 6.64 cases per 10,000 patient-days in 2006 [1]. This increased incidence is comparable to that reported by large hospitals in Canada [2], and mirrors the increases seen in North America and Europe over the past decade. However, national surveillance systems to track CDI rates are relatively less well-developed. Currently, potential surveillance methods include laboratory-based reporting of diagnostic assays or administrative surveillance using International Classification of Diseases, 9^th^ Revision (ICD-9) codes assigned to hospital discharges. Although the primary role of ICD-9 codes is for remuneration, easy accessibility, standardized format across healthcare facilities and consistency over time make ICD-9 codes attractive for surveillance purposes. Previous studies in the United States demonstrated good correlation between toxin assay results and ICD-9 codes, with sensitivity and specificity of ICD-9 codes reported at 71–78% and >99% respectively compared to microbiologic data [3]–[5]. A recent study by Zilberberg and colleagues showed good agreement between pediatric CDI hospitalization rates using administrative (ICD-9) coding from 2 separate databases [6]. However, use of ICD-9 codes for CDI surveillance has not been validated in other healthcare settings including Asia. In this study, we compared CDI rates based on laboratory diagnostic testing to CDI diagnoses captured by ICD-9 codes for hospitalized patients to determine the sensitivity and specificity of ICD-9 codes for use as CDI surveillance.

## Methods

Ethics Statement: The institutional ethics review board for the National Healthcare Group Domain Specific Review Board (NHG DSRB) approved the study prior to initiation (approval number DSRB E/09/008). A waiver of informed consent was specifically requested and granted by the NHG DSRB because the study methods utilized retrospective medical record review on hospitalization records of patients who had been discharged over 2 years before, and the data would be collected in anonymized unlinked datasets and reported in anonymized aggregate form.

The study was conducted at Tan Tock Seng Hospital, a 1200-bed public hospital in Singapore. All patients in 2007 with positive *Clostridium difficile* toxin assay (CDTA) or with discharge summaries containing the ICD-9 code of 008.45 for CDI were retrospectively reviewed. Hospital laboratory testing of CDTA, for both Toxin A and B using ELISA and Immunocard (both from Meridian Bioscience, Inc., Cincinnati, OH, USA) was performed on unformed stool samples based on clinical suspicion of CDI. An in-house evaluation of the Immunocard method compared with PCR method gave a sensitivity of 64%, specificity of 95%, positive predictive value of 78% and negative predictive value of 90% (unpublished data) which is comparable to reported literature [7]. Demographic and hospitalization data were extracted from electronic medical records. The χ^2^ test and Fisher's exact test were used to compare categorical variables. Student's *t* test and the Mann-Whitney *U* test were used to compare continuous variables. All statistical analyses were performed using Stata, version 9.0 (Stata Corp). Corresponding percentages, ORs, and 95% confidence intervals are reported.

## Results

**Figure 1:**
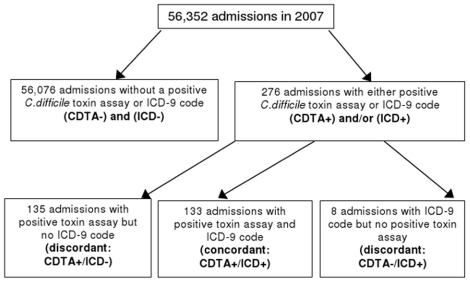
Flowchart of *Clostridium difficile* diagnoses among all patients admitted in 2007 to Tan Tock Seng Hospital.

Of 56,352 admissions to Tan Tock Seng Hospital in 2007, 2,212 (3.9%) patients had CDTA requested. *Clostridium difficile* toxin assay were positive in 268 (12.1%) but only 133 were assigned the ICD-9 code [Figure 1]. An ICD-9 code of 008.45 for CDI was assigned to 141 discharges. Of these, 133 had CDTA-positive and 8 had CDTA-negative results. Review of medical records confirmed symptoms of diarrhea, abdominal pain or cramping in all patients with positive CDTA or ICD-9 code.

**Figure 2:**
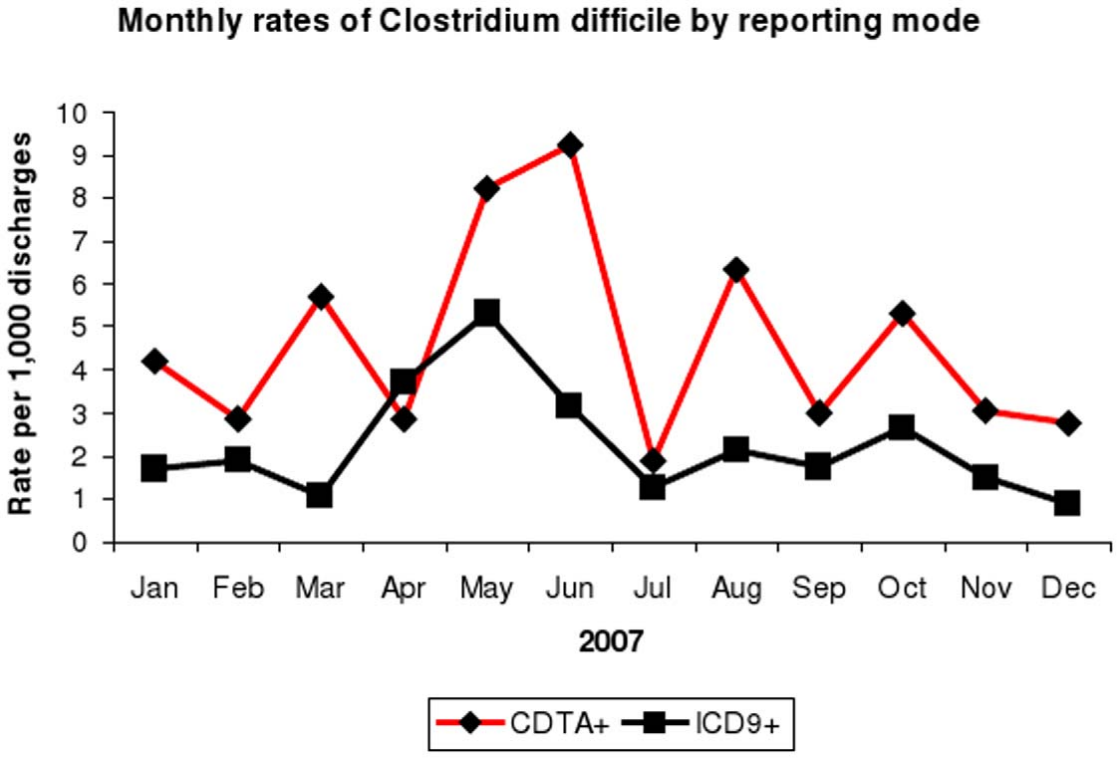
Monthly rates of *Clostridium difficile* by reporting mode.

The sensitivity of ICD-9 codes was 49.6% and the specificity was 99.99%, using laboratory diagnosis as the reference standard. Both positive and negative predictive values of the ICD-9 code were excellent at 94.3% and 99.8% respectively. The CDI rate was 2.50 per 1000 admissions based on ICD-9 codes, compared to 4.76 per 1000 admissions based on laboratory test reporting [Figure 2]. Although CDI rate was under-estimated by use of ICD-9 code, the time series graph showed fair correlation between rates of CDTA and ICD-9 code with good concordance rate (K = 0.649; p<.001).

Length of hospitalization, admitting discipline, ethnic group, and configuration of hospital beds (rooms with 1, 2, 4 or 6 beds) were not significant factors for missing ICD-9 code [Table 1]. Comparing concordant patients with positive toxin assays and ICD-9 codes (CDTA+/ICD9+) to discordant patients with positive toxin assays but no ICD-9 codes (CDTA+/ICD9−), median age was significantly older in concordant patients (75 vs 69 years; p<.001) and median time from admission to positive stool test collection was significantly shorter (8 vs 10 days; p = 0.048) [Table 1]. Concordant patients (CDTA+/ICD9+) were more likely to be over 50 years of age (OR, 3.49; 95% CI, 1.66–7.34; P = .001) and had a shorter time from admission to stool testing (OR, 0.98; 95% CI, 0.97–0.99; P = .009) [Table 2]. Review of the electronic medical record for the discordant cases (CDTA+/ICD9−) revealed 71 of the 135 cases (52.6%) had the diagnosis of CDI discussed in the text of the discharge summaries, but were inadvertently missed by the coders.

**Table 1:** Factors associated with concordant Clostridium difficile diagnosis by positive *Clostridium difficile* toxin assay (CDTA+) and ICD-9 code (ICD9+).

Univariate analysis			
Variables	Group ACDTA+ ICD9− (n = 135)	Group BCDTA+ ICD9+ (n = 133)	p value
Age, median years (range)	69 (16–98)	75 (21–101)	0.0007
Age >50 yrs, n (%)	102 (75.6)	122 (91.7)	0.0004
Male gender, n (%)	82 (60.7)	67 (50.4)	0.09
LOS, median days (range)	22 (1–689)	21 (2–190)	0.549
LOS >30 days, n (%)	59 (43.7)	55 (41.4)	0.697
Time from admission to positive stool collection, median days (range)	10 (0–452)	8 (0–70)	0.048
Admission day to test >30 days, n (%)	30 (22.2)	16 (12.0)	0.027
Test to Date of discharge, median days (range)	11 (0–331)	11 (1–131)	0.216
Surgical discipline, n (%)	12 (9.0)	18 (13.3)	0.263
Chinese, n (%)	100 (74.1)	101 (76.0)	0.724
Malay, n (%)	20 (14.8)	16 (12.0)	0.503
Indian, n (%)	11 (8.2)	12 (9.0)	0.798
Others, n (%)	4 (2.9)	4 (3.0)	0.983
C class, n (%)	81 (60.0)	74 (55.6)	0.469
B1 class, n (%)	4 (3.0)	9 (6.8)	0.147
B2 class, n (%)	43 (31.9)	46 (34.6)	0.635
A class, n (%)	7 (5.2)	4 (3.0)	0.369
B2 or C class (5–6 beds), n (%)	124 (91.9)	120 (90.2)	0.641

**Table 2:** Factors associated with concordant Clostridium difficile diagnosis by positive *Clostridium difficile* toxin assay (CDTA+) and ICD-9 code (ICD9+).

Multivariate analysis	Adjusted OR (95% CI)	p value
Age >50 yrs	3.49 (1.66 to 7.34)	0.001
Time from admission to positive stool collection	0.98 (0.97 to 0.99)	0.009

Among those who had a positive toxin assay, 62 (23%) had their positive stool samples collected within 48 hours of admission. Of these 62, only 2 (3.2%) had no public hospital admissions or government outpatient clinic attendances within the preceding 12 weeks [8]. This suggests that true community-associated CDI without any healthcare facility exposures remains uncommon at this time.

Of the 8 patients who had a positive ICD-9 code but negative CDTA (CDTA−/ICD9+), none had colonoscopy performed for confirmatory histological or pathological diagnosis. However, 7 were considered to have CDI by their treating physicians and received treatment for presumptive CDI.

## Discussion

Rates of *Clostridium difficile* infection would be substantially underestimated by ICD-9 codes compared to laboratory testing in our setting. Sensitivity of ICD-9 codes is poor compared to laboratory testing but specificity remains high. A substantial reduction in sensitivity is attributable to coder interpretation of medical records. More than half of the cases with missed ICD-9 codes had the CDI diagnosis written in the text of the medical discharge summary, yet were not coded appropriately. Inclusion of these records would increase sensitivity from 49.6% to 76%, comparable to other published studies [3]–[5]. Training for coders could improve sensitivity and make ICD-9 coding feasible as a surveillance instrument for CDI. Although ICD9 coding system is widely used and should be standardized, differences in institutional structure and awareness of *Clostridium difficile* may influence coding practices. Public hospitals in Singapore offer government-subsidized healthcare with individual “co-payment” from compulsory medical saving plans. It is possible that differences in payers and billing methods could affect the rigor of medical coding if compared to private hospitals or insurance-based healthcare systems elsewhere. These findings suggest variations in sensitivity that should be validated before use in surveillance.

Previous studies have shown variable sensitivities of ICD-9 codes depending on infection type, varying from 95% in central nervous system infection [8] to as low as 5.9% in sepsis [9], [10]. Use of ICD-9 codes in Clostridium infection has moderate to superior sensitivity of 71–78% compared to other infective conditions [3]–[5], probably due to the well defined signs, symptoms and diagnostic methods for a case definition [11].

Factors associated with concordant results were older age, possibly due to increased awareness of multiple diagnoses related to hospital admission. Shorter time between admission date and ordering of CDTA may again increase likelihood of inclusion of CDI in medical records and subsequent coding. Community-associated CDI cases were rare, with the overwhelming majority having had recent healthcare exposure in the preceding 12 weeks.

Electronic microbiology reporting appears to be a reliable measure for surveillance purposes [12]. Using administrative data did not identify a significant number of additional cases. One advantage of laboratory reporting is the capture of outpatient cases and cases from affiliated healthcare facilities such as rehabilitation centers or nursing homes which may rely upon hospital laboratories for diagnostic testing. An automated algorithm combining microbiology, pharmacy and coding data has become the case-finding method of choice at some institutions [13] although this may have limited advantages compared to microbiology data and incurs increased costs [14].

This study was limited to a single public hospital over a one-year period and our findings may not necessarily apply to other institutions within Singapore or elsewhere. This study on 2007 data may need to be repeated with data from other years in order to determine if and how these findings might vary over time. With *C.difficile* emerging as a public health threat in the past decade, countries seeking to establish national surveillance for CDI may choose administrative data as their surveillance instrument because of relative accessibility, consistency between different datasets and for longitudinal comparisons [6]. Our findings underscore the importance of validating the use of administrative data such as ICD-9 codes for tracking disease trends because reliability from one geographic region or healthcare system may not translate directly to others.

In conclusion, although good sensitivity and specificity were found in US studies for CDI surveillance, exclusive use of ICD-9 codes would substantially underestimate CDI incidence in Singapore. Laboratory reporting would provide a more accurate measure of CDI incidence. Nevertheless, ICD-9 codes correlates moderately well with laboratory reporting for disease trends, and would remain a useful indicator for tracking CDI disease trends for surveillance purposes.
